# Multiple imputation for patient reported outcome measures in randomised controlled trials: advantages and disadvantages of imputing at the item, subscale or composite score level

**DOI:** 10.1186/s12874-018-0542-6

**Published:** 2018-08-28

**Authors:** Ines Rombach, Alastair M. Gray, Crispin Jenkinson, David W. Murray, Oliver Rivero-Arias

**Affiliations:** 1 Health Economics Research Centre, Nuffield Department of Population Health, University of Oxford, Oxford, UK; 2 Nuffield Department of Orthopaedics, Rheumatology and Musculoskeletal Sciences, University of Oxford, Oxford, UK; 3 Health Services Research Unit, Nuffield Department of Population Health, University of Oxford, Oxford, UK; 4 National Perinatal Epidemiology Unit, Nuffield Department of Population Health, University of Oxford, Oxford, UK

**Keywords:** Missing data, Incomplete data, Questionnaires, Randomised controlled trials (RCTs), Quality of life (QoL), Domains

## Abstract

**Background:**

Missing data can introduce bias in the results of randomised controlled trials (RCTs), but are typically unavoidable in pragmatic clinical research, especially when patient reported outcome measures (PROMs) are used. Traditionally applied to the composite PROMs score of multi-item instruments, some recent research suggests that multiple imputation (MI) at the item level may be preferable under certain scenarios.

This paper presents practical guidance on the choice of MI models for handling missing PROMs data based on the characteristics of the trial dataset. The comparative performance of complete cases analysis, which is commonly used in the analysis of RCTs, is also considered.

**Methods:**

Realistic missing at random data were simulated using follow-up data from an RCT considering three different PROMs (Oxford Knee Score (OKS), EuroQoL 5 Dimensions 3 Levels (EQ-5D-3L), 12-item Short Form Survey (SF-12)). Data were multiply imputed at the item (using ordinal logit and predicted mean matching models), sub-scale and score level; unadjusted mean outcomes, as well as treatment effects from linear regression models were obtained for 1000 simulations. Performance was assessed by root mean square errors (RMSE) and mean absolute errors (MAE).

**Results:**

Convergence problems were observed for MI at the item level. Performance generally improved with increasing sample sizes and lower percentages of missing data. Imputation at the score and subscale level outperformed imputation at the item level in small sample sizes (*n* ≤ 200). Imputation at the item level is more accurate for high proportions of item-nonresponse. All methods provided similar results for large sample sizes (≥500) in this particular case study.

**Conclusions:**

Many factors, including the prevalence of missing data in the study, sample size, the number of items within the PROM and numbers of levels within the individual items, and planned analyses need consideration when choosing an imputation model for missing PROMs data.

**Electronic supplementary material:**

The online version of this article (10.1186/s12874-018-0542-6) contains supplementary material, which is available to authorized users.

## Background

Missing data can introduce bias in the results of randomised controlled trials (RCTs), which can have a negative impact on clinical decisions derived from them, and ultimately patient care. Patient reported outcome measures (PROMs), which are increasingly used in RCTs as primary or key secondary endpoints [1, 2], can be particularly susceptible to containing missing data, either due to unasnwered or incomplete questionnaires [3, 4]. PROMs are carefully designed and validated instruments, often in questionnaire form, intended to capture information on health status from the patients’ perspective [5–7]. The majority of PROMs consists of several questions, or items, and are hence referred to as multi-item PROMs. The PROMs items are usually combined into one composite score and/or subscales. Missing data in multi-item PROMs can occur either in the form of unit-nonresponse, where all items have been left unanswered [8], or item-non-response, where responses to the PROM are incomplete [9]. Missing data can affect the calculation of the composite score and/or subscales. Some scoring manuals allow for small amounts of missing items, while other scoring manuals do not facilitate the calculation of composite scores in the presence of any missing items.

Traditionally, research concerning missing data in PROMs has focussed on how the missing PROMs composite scores should be handled, with multiple imputation (MI) methods considered to be one of the most reliable methods [10–12], although MI is not commonly implemented in the analysis of RCT data [13–16]. However, for multi-item PROMs, different imputation approaches are possible, e.g. imputation at the composite score, subscale (where available) or items level. Imputation at the item or subscale level may yield additional information and therefore improve the accuracy of such imputations.

Research has not commonly been performed on the comparison between these approaches. Work by Simons et al. [17] compared imputation at the item and composite score level for estimating EuroQoL 5 Dimensions 3 Levels (EQ-5D-3L) composite scores in the presence of missing at random (MAR) data. The authors found that both approaches performed similarly in terms of accuracy for larger data sets (*n* > 500) and where missing data primarily followed a unit-nonresponse pattern for all different proportions of missing data investigated (i.e. 5–40% of missing data). As the sample size was decreased to 500 observations or fewer, both approaches performed similarly for up to 10% of missing data, however, MI at the composite score level was found to be more accurate for 20 and 40% of missing data within these smaller sample sizes. MI at the item level was found to be performing better as the proportion of item-nonresponse increased. The authors recommended further research to assess generalizability of their findings to other PROMS with potentially different psychometric properties.

Eekhout et al. [18] compared a number of different methods to account for missing data in the Pain Coping Inventory (PCI), a 12-item PROM. In their work, the PCI was used as a covariate in a regression model, and the different MI approaches were compared in terms of accuracy and precision of the fitted PCI regression coefficients. In this scenario, MI at the item level achieved the best results, while MI applied to the composite scores resulted in overestimated standard errors where large percentages (> 50%) of participants had missing data. The authors also found that complete cases analysis (CCA), which does not impute missing data, yielded acceptable results in terms of regression coefficients. However, standard errors were overestimated, especially when more than 10% of the study population had some missing PROMs data, and therefore the authors advised against the use of CCA. However, other research has suggested that CCA may be appropriate for the analysis of RCTs under specific circumstances, i.e. when missing data is limited to a single outcome and if the variables in the MAR mechanism are included in the covariates in the analysis model [19].

### Hypotheses for this work

The composite scores, and subscales where applicable, for many multi-item PROMs are calculated as the unweighted [20] or weighted [11, 12] sum of the items. Generally, composite scores cannot be derived if at least one item is missing, although some scoring manuals allow for a small number of items to be substituted by the mean score of the available items [20, 21].

All items contribute to the calculation of the composite scores. Therefore, we hypothesise, similarly to Simons et al. [17], that where the MAR data follow an item-nonresponse pattern, imputation at the item level is superior to that at the composite score or subscale level, particularly as the proportion of item nonresponse increases, as the latter approaches disregard some of the available data. Correspondingly, we hypothesise that where the MAR data follow primarily a unit-nonresponse pattern, all MI approaches perform similarly, as in this scenario the MI at the item level cannot utilise any additional information that is not available to the MI at the composite score or subscale level. Where validated subscales exist for a PROM, we hypothesise that there are benefits in terms of accuracy when imputing at the subscale level compared to imputing at the composite score level, given sufficient data is available to calculate at least one of the subscales. No benefit of applying MI at the subscale level over MI at the composite score level is expected in unit-nonresponse scenarios, or where neither of the subscales can be estimated due to missing data.

CCA is expected to perform similarly to MI at the composite score level under MAR for appropriately adjusted analysis models, i.e. where the covariates include the key components of the MAR mechanism [19].

### Aims of this research

This research aims to compare different MI approaches for handling missing PROMs data, i.e. imputation at the composite score, subscale (where appropriate) or item level, while also exploring the benefits and disadvantages of these approaches. A variety of different MAR patterns, sample sizes and proportions of missing data are explored using simulation studies using three widely used PROMs. Performance of the different imputation approaches in terms for producing composite scores and adjusted treatment effects are considered, together with the performance of CCA for the generation of treatment effects. This research aims to validate previous findings in a different dataset, and to expand this research to additional PROMs. This work will generate clearer guidelines for the appropriate handling of missing PROMs endpoints, specifically with regards to the use of MI.

## Methods

### Design of the simulation exercise

**Fig. 1:**
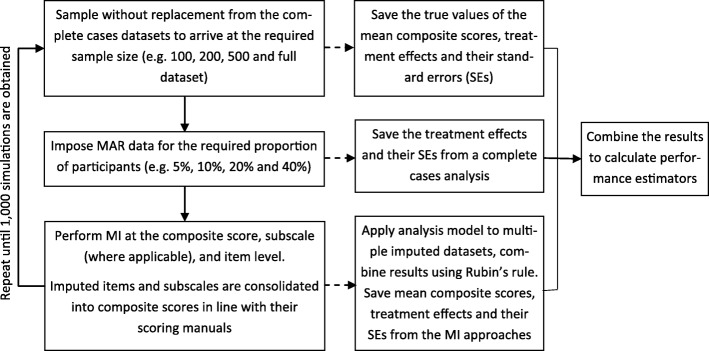
Design of the simulation study. MAR – missing at random, MI – multiple imputation, SE – standard error, RMSE – root mean square error, MAR – mean absolute error

An overview of the simulation study is provided in Fig. 1. Simulations started with a complete dataset of the relevant sample size. A pre-specified proportion of MAR data was then introduced in the PROMs data at follow-up. MI was performed at the composite score, subscale (where applicable) and item level. Estimates of the mean composite scores, treatment effects and corresponding standard errors (SE) were obtained from the complete dataset (i.e. the ‘true’ estimates) and the different MI approaches. In addition, the treatment effect was also estimated from the dataset with imposed missing data using a CCA, i.e. an analysis that excludes all participants with missing outcome data. The treatment effects were estimated using a regression model with the relevant composite PROMs score as the outcome variable adjusting for baseline composite scores, randomisation allocation, age and sex [22].

This simulation study aimed to obtain 1000 independent iterations for which the imputation models successfully converged (i.e. 1000 valid imputation results) for each scenario. Where imputation models did not converge, additional iterations were run (up to a maximum of 11,000 iterations per scenario).

The parameters of interest in this simulation study are the mean composite outcome scores and the average treatment effect. The performance parameters used in this study were root mean square errors (RMSE), mean absolute errors (MAE – shown in Additional file 1).

The simulation work was performed in StataSE 14 [23], and the *mi impute and mi estimate* commands were used.

### Case study

This simulation study was based on data collected within the KneeArthroplasty Trial (KAT) [22, 24]. KAT is a large multi-centre RCT considering the clinical and cost effectiveness of new developments in knee replacements. KAT was designed as a partial factorial, pragmatic trial, with participants being randomised to at least one of four different comparisons. In this simulation study, only one of the comparisons is considered, i.e. patellar resurfacing vs. no patellar resurfacing. Long-term follow-up beyond 10 years is ongoing for KAT. Here, data for a single follow-up time point at 5 years post randomisation were considered, and only participants with fully observed baseline and outcome data were included in the simulation study.

A total of 1715 participants were randomised to the patellar resurfacing vs. no patellar resurfacing comparison. In this simulation study, the 11% of participants known to have died before the 5 year follow-up were excluded. Of the 1526 remaining participants, 5 year follow-up data for the Oxford Knee Score (OKS), EQ-5D-3L and 12 item Short Form Survey (SF-12) were unavailable for 17, 18 and 33%, respectively, with missing data rates similar in both treatment arms. The OKS can be calculated for up to two missing items, and there are additional participants (approximately 7%) for whom one or two items are missing. One thousand four hundred twenty-two participants were eligible to complete version 2 of the SF-12. The remaining 104 participants completed version 1 of the SF12 at a minimum of one point in the trial and were not included in these summaries.

Correlations between the PROMs (composite scores, subscales and items) and the baseline data to be used in the imputation and analysis modes are shown in the Additional file 1. The correlations are low to moderate, which is common in RCT datasets.

### Instruments

The 5 year follow-up data for three patient reported outcome measures is used, namely:

- The Oxford Knee Score (OKS): an instrument designed to assess outcomes following a knee replacement in RCTs [25, 26]. It consists of 12 five-level items, and the composite score ranges from 0 to 48. The OKS can be divided into validated pain and function subscales [20]. Higher scores indicate better outcomes.
- The SF-12: a 12-item generic health measure [27, 28]. The SF-12 generates two subscales, the physical component summary score (PCS) and the mental health component summary score (MCS). Both subscales are standardised to have a mean of 50 with a standard deviation of 10 [29]. As the calculations for both the MSC and PCS utilise all items, rather than just a subset, they are referred to as ‘composite scores’ subsequently for consistency. Higher scores indicate better outcomes.
- EQ-5D-3L: a utility questionnaire assessing participants’ health state based on their mobility, self-care, usual activities, pain/discomfort and anxiety/depression [30]. Scores of 1 indicate full health, 0 indicates a health state equal to death, and scores lower than 0 indicate health states worse than death.

### Missing data simulation

**Fig. 2:**
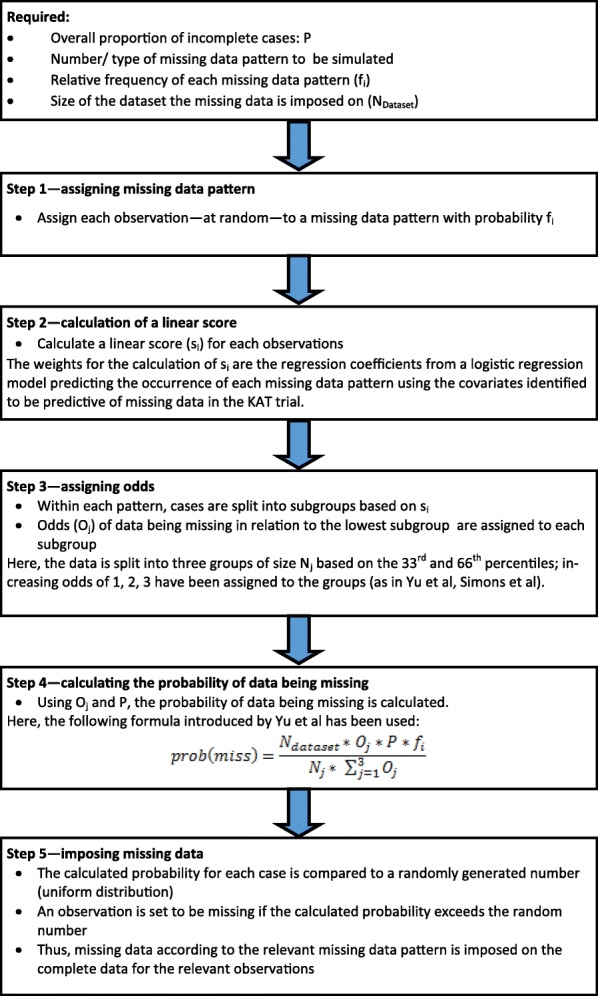
Depiction of the algorithm used for each iteration simulation of missing PROMs data within the complete cases dataset

Missing data were introduced in a subset of participants with completely observed data for their PROMs outcomes and relevant baseline data (*N* = 1030 for the OKS, *N* = 1160 for the EQ-5D-3L and *N* = 797 for the SF-12 MCS and PCS – i.e. the ‘base cases’). The simulated missing data patterns mirrored those most commonly observed at the selected follow-up for the three PROMs, which followed predominantly a unit-nonresponse pattern (Table 1). For the OKS, additional missing data patterns were simulated, i.e. a scenario where all missing data was due to unit-nonresponse, as well as a scenario where 70% of the missing data was due to item-missingness. MAR data was simulated using an algorithm by van Buuren et al. [31] and also outlined in publications by Yu et al. [32] and Simons et al. [17]. This algorithm allows researchers to vary the missing data patterns, as well as the percentage of participants with missing data; implementation followed the steps outlined in Fig. 2. Missing data was generated for 5, 10, 20 and 40% of participants. Sample sizes of 100, 200, 500 and the maximum sample available were considered in this simulation work. Smaller sample sizes were obtained by sampling the required number of participants from the full dataset without replacement prior to the simulation of missing data.

**Table 1:** Missing data patterns simulated for each PROM

OKS	OKS	SF-12	SF-12	EQ-5D-3L	EQ-5D-3L
OKS missingness patterns	Observed missing data pattern	SF-12 missingness patterns	Observed missing data pattern	EQ-5D-3L missingness patterns	Observed missing data pattern
Unit non-response	73.1%	Unit non-response	56.1%	Unit non-response	87.9%
Only item 7 missing	15.6%	Only item 2b missing	20.3%	Only item 5 missing	5.1%
Only item 4 missing	3.3%	Only item 4b missing	6.5%	Only item 1 missing	2.6%
Only item 6 missing	2.7%	Items 2b and 3b missing	4.5%	Only item 4 missing	1.8%
Only item 9 missing	2.1%	Only item 3b missing	4.0%	Only item 3 missing	1.5%
Only item 10 missing	1.5%	Items 2b, 3b and 4b missing	3.5%	Only item 2 missing	1.1%
Only item 1 missing	0.9%	Items 2b and 4b missing	3.3%	n/a	Other patterns occurred too infrequently to be used in simulation
Only item 12 missing	0.9%	Only item 6c missing	1.8%	n/a

Variables included in the algorithm to generate MAR data were treatment allocation, age, baseline PROMs score, height, ASA Grade (American Society of Anesthesiologists physical status classification system) and size of the recruiting centre (< 30, 30–100, > 100 participants).

### Implementation of the MI models

All imputation models were implemented using an MI by chained equations (MICE) approach [33]. MI models to handle missing continuous data (i.e. composite scores and subscales) were based on linear regression models, using a predicted mean matching (PMM) approach. Imputations at the item level were implemented as ordered logistic regression models and also as regression models (using PMM), treating the items as continuous variables. Covariates used in all MI models included the baseline composite PROM score, as well as all variables used in the analysis model and those used in the simulation of MAR data. For the OKS simulations, MI models at the subscale and item level also included the baseline values of the subscales.

Imputations were performed separately by randomised treatment, where feasible [19]. This approach allows factors such as the distribution of outcomes, their variance and relationship with any of the covariates to differ between treatment arms. If model convergence was low, imputations included the randomised treatment as a covariate instead. The number of imputations was 50 for all imputations at the composite score and subscale level. For MI at the item level and simulations reproducing the observed missing data patterns, 50 imputations were run for the base cases, and imputations equal to the percentage of missing data were used for smaller sample size [33], while 10 imputations were used for exploratory scenarios.

The MI models at the item level were complex, and convergence issues have been demonstrated [17]. Therefore, imputations at the item level were run one-by-one (i.e. using the *add(1)* option in Stata’s *mi impute* command), which ensured that additional imputations continued to be generated after one out of the required imputations failed. We allowed for up to 50% of the required imputations to fail to converge before an iteration of the item level imputation simulation was classed as having failed.

After the presentation of the simulation results, the different imputation approaches were applied to a case study to examine how they affected the interpretation of the trial. This example included a random subset of 200 participants, with missing data imposed as described above for approximately 20% of participants.

## Results

### Feasibility of the MI approaches

Imputations at the composite score and subscale level were feasible in all simulation scenarios. However, convergence failures were observed for almost all imputations at the item level using the ologit approach; instances of non-convergence increased markedly for decreasing sample sizes and increasing proportions of missing data (Table 2). For this reason, item-level imputations for the OKS and SF-12 were not run separately by treatment arm as a compromise. Considerations for this approach are discussed later. Scenarios with fewer than 1000 valid imputations at the item level were not included in subsequent comparisons, e.g. insufficient valid results were obtained for MI at the item level for 10% or more missing data for sample sizes of 100, and for the combination of 40% of missing data and a sample size of 200.

**Table 2:** Proportion of item-level simulations (ologit) not converging until 1000 valid simulation results were obtained*

Sample size	Proportion of missing data	OKS			SF-12	EQ-5D-3L
		Observed missing data pattern	Unit-non-response	70% item missingness	Observed missing data pattern	Observed missing data pattern
100	5	88.2%	89.7%	88.0%	91.9%*	72.1%
	10	95.6%*	95.3%*	94.1%*	98.2%*	83.8%
	20	99.8%*	99.6%*	99.1%*	99.9%*	94.4%*
	40	100%*	100%*	100%*	100%*	99.9%*
200	5	40.1%	22.7%	35.4%	52.1%	19.9%
	10	50.8%	25.2%	45.6%	69.6%	25.5%
	20	64.5%	32.1%	57.0%	87.2%	45.30%
	40	99.7%*	60.0%	78.4%	99.3%*	85.6%
500	5	21.6%	8.84%	20.0%	3.5%	11.2%
	10	24.9%	9.3%	23.4%	6.3%	14.2%
	20	27.8%	11.0%	27.5%	12.8%	17.3%
	40	41.4%	16.0%	28.6%	34.7%	23.6%
Full sample^a^	5	1.0%	0%	0.7%	0%	0.3%
	10	3.9%	0.1%	1.6%	0.2%	1.86%
	20	12.5%	0.4%	5.2%	1.1%	6.8%
	40	28.0%	3.8%	10.4%	8.4%	17.4%

*For scenarios highlighted *, 1000 valid simulations could not be obtained, and these scenarios are not included in subsequent summaries

^a^The full sample includes 1030 observation for the OKS simulation, 797 for the SF-12 and 1160 for the EQ-5D-3L

Some instances of non-convergence were also observed for small sample sizes for imputation at the item level using the PMM approach.

### Performance of the different imputation approaches and CCA

Generally, RMSE (and MAE; see Additional file 1) increased with increasing percentages of missing data, as well as with decreasing sample size.

#### OKS with observed missing data pattern

**Fig. 3:**
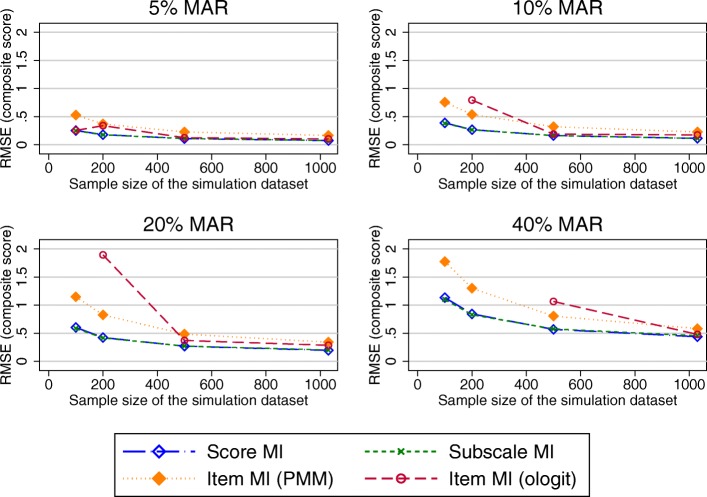
RMSE in the OKS composite score estimates (observed missing data patterns). Abbreviations: MAR – Missing at random; MI – Multiple imputation; OKS – Oxford knee score; PMM – Predicted mean matching; RMSE – Root mean square error

Figure 3 shows the RMSE in the estimated OKS composite scores after applying the different MI approaches to datasets covering a range of sample sizes and proportions of participants with missing PROMs data following the observed missing data pattern.

**Fig. 4:**
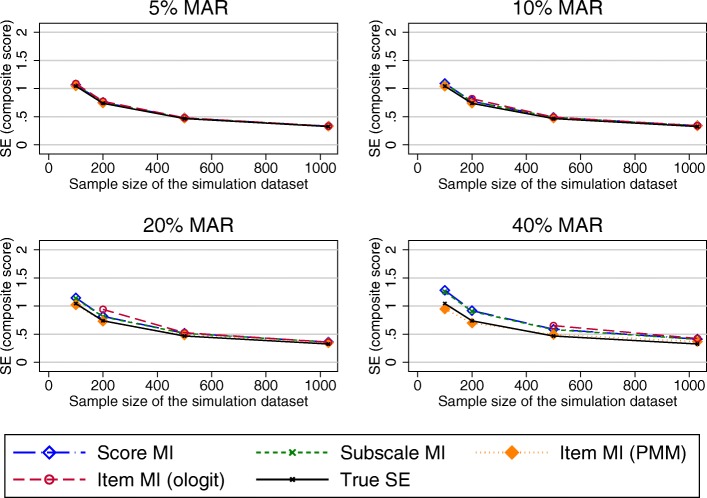
SE in the OKS composite score estimates (observed missing data pattern). Abbreviations: MAR – Missing at random; MI – Multiple imputation; PMM – Predicted mean matching; SE – Standard Error

RMSE for MI at the composite score level was almost identical to the RMSE for MI at the subscale level. Higher levels of the RMSE were observed for MI at the item level (both the ordinal logit and PMM approach) for higher proportions of missing data and smaller sample sizes. The SEs for these composite score estimates were larger than the true SE for scenarios with 20 and 40% of MAR data for all imputation approaches (Fig. 4).

**Fig. 5:**
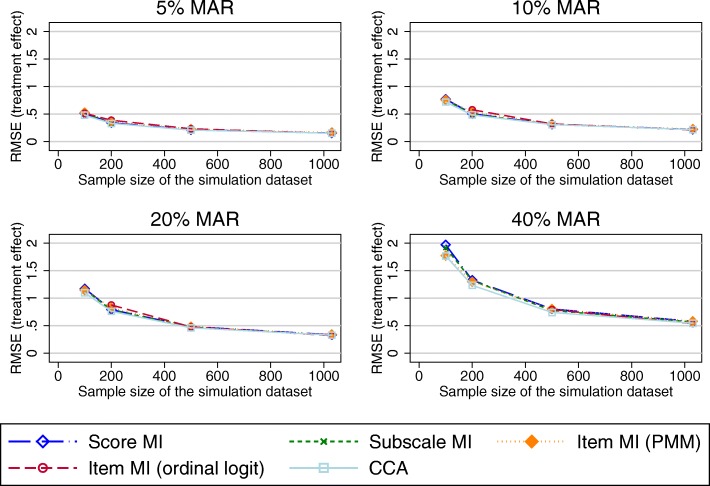
RMSE in the treatment effect estimates using the imputed OKS composite scores as the outcome variable in the regression model (observed missing data pattern). Abbreviations: CCA – Complete cases analysis; MAR – Missing at random; MI – Multiple imputation; OKS – Oxford knee score; PMM – Predicted mean matching; RMSE – Root mean square error

Figure 5 presents the RMSE in the treatment effect estimates using the imputed OKS composite scores as the outcome variable in the regression model. All MI approaches and CCA performed very similarly. As above, the SE for these estimates was marginally increased compared to the true SE for scenarios of 20% or more MAR data.

#### Considering other missing data patterns for the OKS

**Fig. 6:**
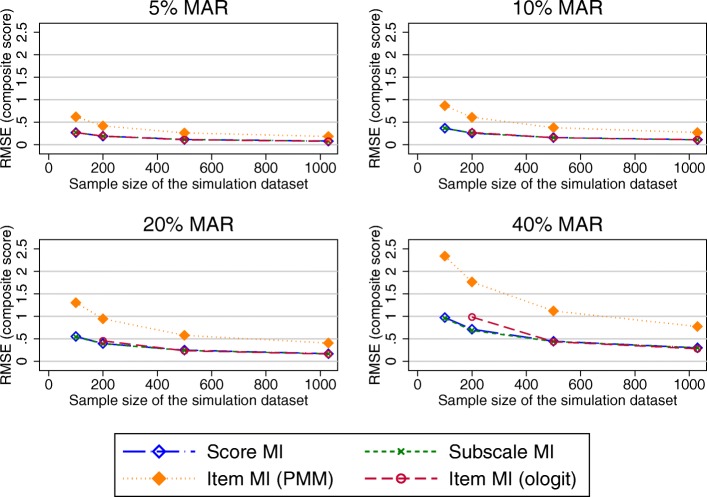
RMSE in the OKS composite score estimates (unit-nonresponse). Abbreviations: MAR – Missing at random; MI – Multiple imputation; OKS – Oxford knee score; PMM – Predicted mean matching; RMSE – Root mean square error

Under a unit-nonresponse scenario, the RMSE observed in the composite scores estimates was similar for all imputation at the composite score, subscale and item level using the ologit approach, except for small sample sizes and 40% of missing data, where performance of the item level MI was marginally worse (Fig. 6). Imputation at the item level using a PMM approach performed worse. MI approaches and the CCA performed similarly in terms of bias observed in the treatment effects, except for imputation at the item level using a PMM approach, which had higher RMSEs for larger proportions of missing data.

**Fig. 7:**
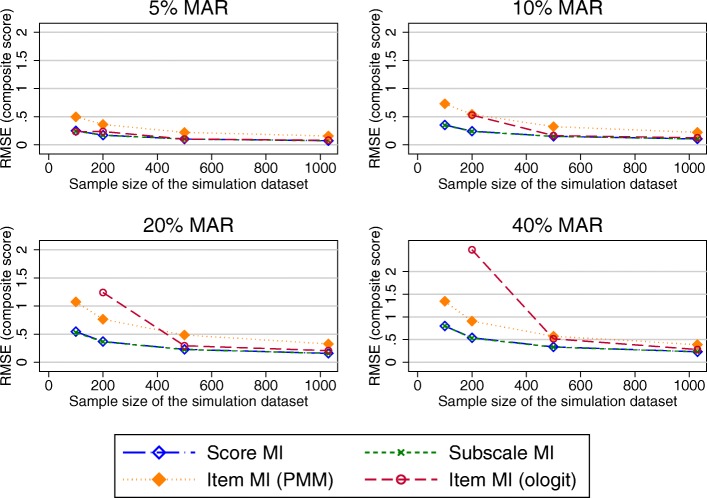
RMSE in the OKS composite score estimates (70% item non-response simulations). Abbreviations: MAR – Missing at random; MI – Multiple imputation; OKS – Oxford knee score; PMM – Predicted mean matching; RMSE – Root mean square error

**Fig. 8:**
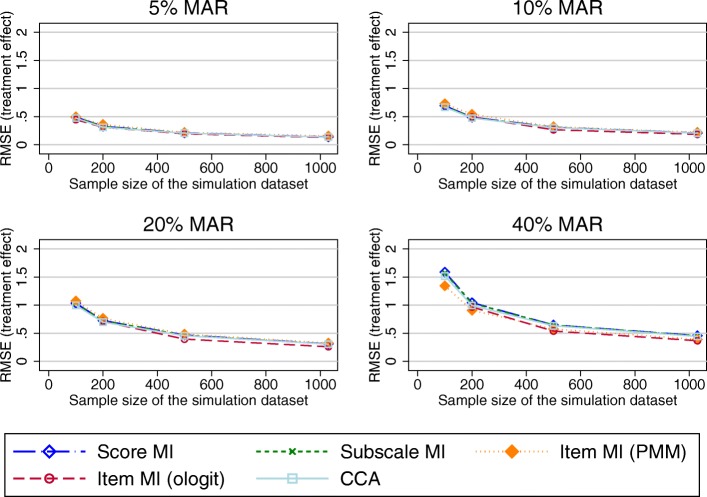
RMSE in the treatment coefficient estimates using the imputed OKS composite scores as the outcome variable in the regression model (70% item-nonresponse). Abbreviations: CCA – Complete cases analysis; MAR – Missing at random; MI – Multiple imputation; OKS – Oxford knee score; PMM – Predicted mean matching; RMSE – Root mean square error

When the item-nonresponse was increased to 70%, all approaches performed similarly for large sample sizes. Item imputation (both ologit and PMM approaches) performed worse than its comparators for small sample sizes when composite scores were estimated (Fig. 7). Considering the estimates of the treatment effects, imputation at the item and subscale level offered a marginal benefit over imputation at the composite score level and CCA in terms of the performance measure observed in the treatment effects for large proportions of missing data (20% or more), as seen in Fig. 8. The different approaches of handling missing data performed similarly for smaller proportions of missing data.

#### SF-12 simulation results

**Fig. 9:**
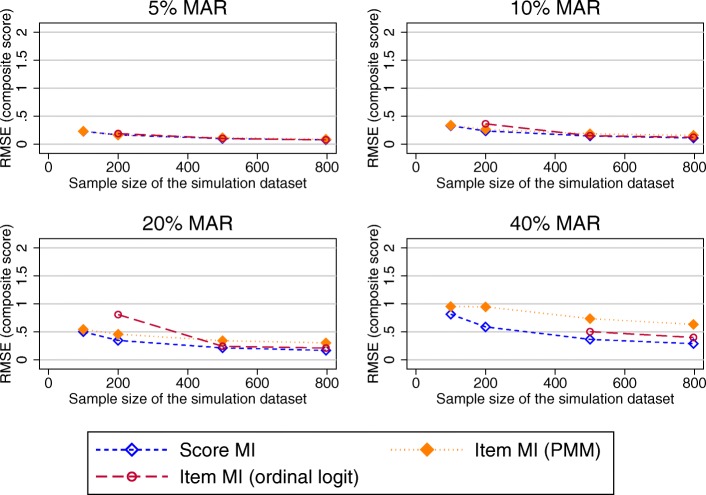
RMSE in the MCS composite score estimates. Abbreviations: MAR – Missing at random; MI – Multiple imputation; MCS – SF-12 Mental component summary score; PMM – Predicted mean matching; RMSE – Root mean square error

Simulated missing data in the SF-12 was due to item missingness for almost 45% of participants. RMSEs observed in the physical component summary score (PCS) were very similar for imputations at the composite score and item-level imputation (ordinal logit), but higher for the item level imputatoion using the PMM approach. For the mental health component summary score (MCS), higher RMSEs were produced by MI at the item level (ologit approach), for combinations of higher proportions of missing data and smaller sample sizes (Fig. 9), with similar results observed otherwise. Imputation at the item level using the PMM approach produced higher RMSEs for higher proportions of missing data.

**Fig. 10:**
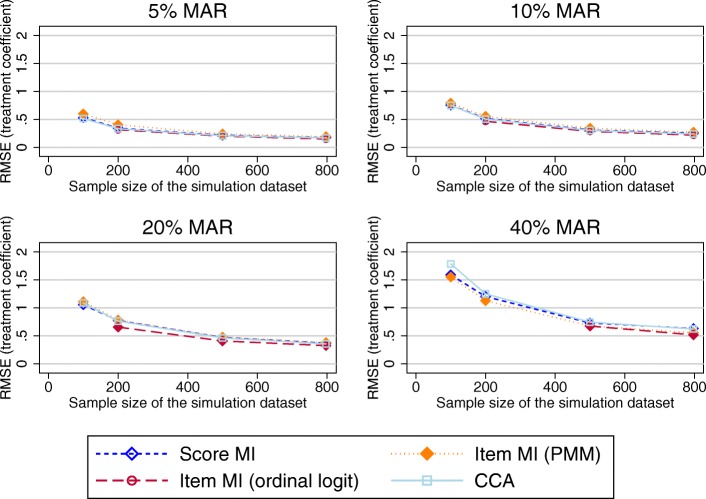
RMSE in the treatment coefficient estimates using the imputed PCS composite scores as the outcome variable in the regression model. Abbreviations: CCA – Complete cases analysis; MAR – Missing at random; MI – Multiple imputation; PCS – SF-12 Physical component summary score; PMM – Predicted mean matching; RMSE – Root mean square error

RMSEs observed in the treatment effects were marginally lower when item imputation was used for combinations of larger proportions of missing data. MI at the composite score level and CCA performed similarly (Fig. 10 –shown for the PCS).

All approaches to handling missing data resulted in similar increases in the SE.

#### EQ-5D-3L simulation results

**Fig. 11:**
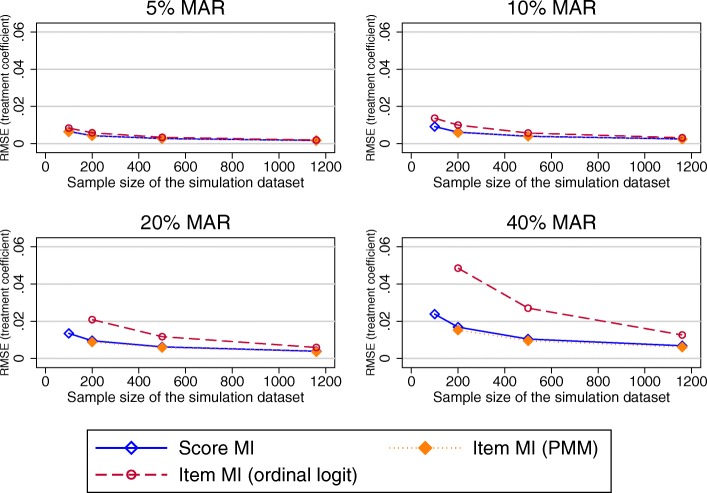
RMSE in the EQ-5D-3L composite score estimates. Abbreviations: EQ-5D-3L - EuroQol 5 dimension 3-level questionnaire; MAR – Missing at random; MI – Multiple imputation; MCS – SF-12 Mental component summary score; PMM – Predicted mean matching; RMSE – Root mean square error

**Fig. 12:**
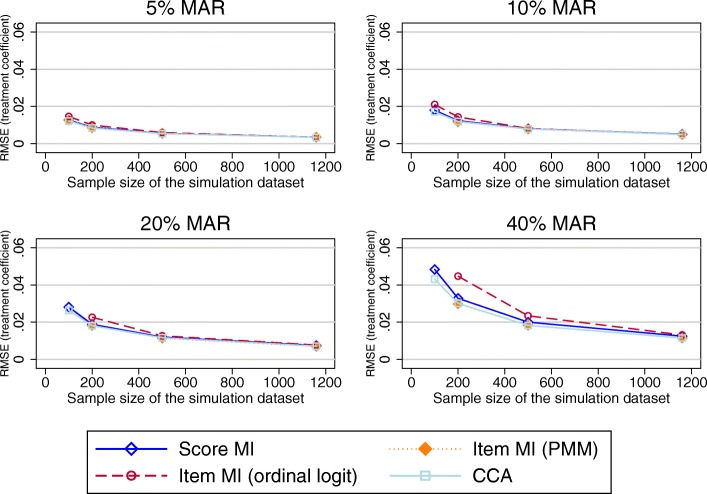
RMSE in the treatment coefficient estimates using the imputed EQ-5D-3L composite scores as the outcome variable in the regression model. Abbreviations: CCA – Complete cases analysis; EQ-5D-3L - EuroQol 5 dimension 3-level questionnaire; MAR – Missing at random; MI – Multiple imputation; PMM – Predicted mean matching; RMSE – Root mean square error

Almost 88% of the missing EQ-5D-3L data was due to unit nonresponse. Considering estimates of the composite scores (Fig. 11), imputation at the composite score level and at the item level using the PMM approach performed similarly. Imputation at the item level using the ologit approach performed worse for combinations of large proportions of missing data and smaller sample sizes. A similar trend was observed for the estimated treatment effects (Fig. 12), although the differences in the RMSE between the item level imputation using the ologit approache and the other imputation approaches were less pronounced. The CCA performed similarly to MI at the composite score level. All approaches performed similarly for large sample sizes.

Additional results using the MAE confirmed the findings reported here and can be accessed in the Additional file 1.

### Application of the different imputation approaches to a case study

The case study includes 200 participants, equally split between the treatment arms, with 17% of participants having missing OKS outcome data. The estimates of the treatment effect are displayed in Table 3. The data shows that the estimates produced from the imputation at the item level (ologit) are most similar to the CCA, while the treatment effect from the imputation at the item level using the PMM approach, subscale and composite levels are very similar, and slightly lower than the estimates produced by the other approaches. However, in this case, all analyses approaches lead to the same conclusion, i.e. that there is insufficient evidence to suggest a statistically significant difference in OKS outcomes between the trial arms.

**Table 3:** Impact of the different analysis approaches on the trial conclusion

Analysis approach	Treatment effect (95% CI)	Number of participants included
Complete cases analysis	0.9 (−2.6, 4.4)	167
MI at the composite score level	0.7 (−2.8, 4.2)	200
MI at the subscale level	0.7 (− 2.7, 4.1)	200
MI at the item level (ologit)	0.9 (−2.5, 4.3)	200
MI at the item level (PMM)	0.6 (−2.7, 3.9)	200

## Discussion

PROMs are commonly used in clinical research, but can be prone to missing data. Analytical methods to limit the potential bias introduced by missing data are widely discussed in the current literature, with MI considered to be one of the most appropriate methods to handle missing data. In this simulation work, MI was not shown to perform significantly better than CCA in estimating treatment effects, in line with the literature [22]. However, this is due to the fact that the variables in the analysis model were also very influential in the algorithm generating the MAR data. In reality, it is likely that the MAR mechanism will be related to more variables outside the analysis model, and hence MI may be preferable to CCA due to its ability to account for complex MAR mechanisms.

When implementing MI, there is little guidance on whether imputation should be applied at the composite score, subscale or item level. This simulation work has shown that MI at the item level may not be feasible for small sample sizes, particularly as the number of PROMS items increases. However, where feasible, it can have advantages over imputation at the composite score level, or subscale level, where applicable, in term of accuracy of the statistical output or facilitation of subsequent analyses. Arguably, the differences in performance for the imputation approaches were relatively small, e.g. up to one point on the OKS, which ranges from 0 to 48, and small differences in the estimated treatment effects, as shown in the case study. These differences lie within the measurement error of the PROM, and do not exceed the minimal important difference, which are estimated to be four points and five points, respectively [34]. However, many trials aim to detect small effect sizes; i.e. the KAT study was powered to detect a difference of 1.5 points in the OKS (patella resurfacing comparison) [22]. Therefore, even these moderate differences could affect trial conclusions, and the choice of MI approach hence needs to be considered carefully, taking into account a multitude of factors:

### Sample size, proportion of missing data and missing data patterns

The different imputation approaches yielded similar results for large sample sizes, as well as smaller sample sizes with 10% missing data or less, except for simulations with high proportions of item-nonresponse, where imputation at the item or subscale level (OKS) may be advantageous. For smaller samples with large amounts of missing data, imputation at the composite score level is likely to be more beneficial in scenarios with a predominantly unit-nonresponse pattern, in line with previous research [17]. Imputation at the item or subscale level (where available) becomes more beneficial with increasing amounts of item-non-response.

Generally, imputation at the item level may not be feasible for scenarios with small sample sizes.

### Feasibility of the imputation model

Item-level imputation models are complex, and may not be feasible for small sample sizes and/or larger proportions of missing data, and the issue of non-convergence becomes increasingly prominent with increasing amounts of items to be imputed, and lower counts in some of the item levels. Using treatment as an explanatory variable in the MI model instead of running imputations separately by treatment arm may enable convergence of complex imputation models at the item level when imputing using ordinal logit models for the PROM items. However, this approach is not in line with current guidance and its appropriateness is dependent on distributional assumptions [19, 35], which need to be assessed on a case-by-case basis. The approach was used in this simulation study as a compromise to achieve higher convergence rates, and justifiable because the distribution of outcomes, their variance and relationship with other covariates was assumed to be the same across treatment arms. However, researchers should bear in mind that this assumption may not hold for other datasets.

Non-convergence of the item imputation using ordinal logit models was caused by perfect prediction, due to very low numbers of observations in one or more of the levels in one of the items to be imputed. Therefore, before attempting imputation at the item level using ordinal logit models, the dataset should be investigated thoroughly for low count and potential problems due to perfect prediction. Even if there is no perfect prediction in the data for the imputation model for a specific item, low counts may still result in unreliable estimates and standard errors to be produced by the statistical models, which are likely to lead to bias in the MI estimates. Therefore, ahead of implementing imputation models, each statistical model to be used in the imputation should be run individually, and separately (by treatment arm if imputations are to be run this way), to confirm that appropriate estimates can be produced.

For combinations of small sample sizes and larger proportions of missing data, problems were also observed for imputation at the item level were a regression model using PMM was used to impute missing data. These issues were related to overfitting of the model, i.e. the use of too many covariates for small amounts of data. Again, the individual statistical models should be checked ahead of implementing any imputation models.

### Planned analysis

While the different imputation approaches may not offer distinct benefits in terms of reducing the RMSE and MAR in some circumstances, there may still be situations where imputation at the item or subscale level is advantageous. This is true where the planned analysis includes not only the analysis of the composite scores, but also of the subscales (where applicable) or even the PROMs items. If feasible, imputation at the item or subscale level ensures that a common imputation dataset can be used for all analyses related to the relevant PROM.

Overall, performance of the analysis approaches decreased with increasing proportions of missing data, emphasising the importance of preventing the occurrence of missing data prospectively [36].

#### Strengths and limitations

This research contributes to the literature in that it uses new datasets to validate previous work on the effect MI at the item and composite score level on the RMSE and MAR observed in the composite scores [17] and treatment effects [18] in PROMS analysis to different datasets and patient populations. In addition, previous research has been extended to additional questionnaires, and additional missing data scenarios, thus offering additional guidance to researchers faced with missing PROMs data in RCTs. This study covers a range of sample sizes (100 to approximately 1000) and rates of missing data (5, 10, 20 and 40%), which are representative of current figures observed in published RCTs [14–16, 37, 38]. While RCTs with lower sample sizes are also common, these are often pilot and feasibility studies which focus on endpoints such as recruitment and completeness of endpoints, or are underpowered for the type of analyses used in this simulation study.

Although every effort was made to conduct this simulation study as thoroughly and completely as possible, it is not without limitations. Scenarios considered are limited to specific sample sizes, proportions of missing data and missing data patterns. However, we believe that sample sizes between 100 to around 1000 participants, and missing data levels between 5 and 40% are representative for the vast majority of RCTs. Future work on larger sample sizes, expanding the generalisability to larger-scale epidemiological research, is needed. These studies often collect a larger pool of patient demographics, the inclusion of which may affect the performance of the imputation models. Similarly, the missing data patterns used were based on those observed in the KAT trial. It is believed that these patterns are realistic and representative for the PROMs used, and we included variations in the amount of unit-nonresponse for the OKS simulations.

This simulation work is restricted to the KAT dataset. Additional validation work in further datasets, other disease areas, as well as PROMs may be useful to explore if the recommendations provided here still hold when the different approaches are applied to datasets with different correlations between baseline and outcome data, distributions of outcomes, different treatment mechanisms and different MAR patterns. However, the fact that findings by Simons et al. [17] and Eekhout et al. [18] could be replicated indicates that findings are generalisable. The main body of this simulation work used the missing data pattern observed in the KAT study at the 5 year follow-up. Additional missing data patterns (i.e. unit non-response and increased levels of item missingness) were only considered for the OKS, in order to supplement the findings by Simons et al. [17] on the effect of increased proportions of unit-non-response in the EQ-5D-3L.

Findings are limited to PROMs with up to 12 items. Therefore, uncertainty still exists as to the maximum number of items within a PROM for which item imputation would still be considered feasible, which is likely to be related to both the construct of the PROM, as well as the sample size. However, we believe that larger datasets are needed to ensure feasibility of item imputation for PROMs with more than 12 items, which are therefore not within the remit of this research.

In this simulation work, the relative performance of the imputation approaches appeared to be related to the outcome of interest, with smaller differences for the estimation of the treatment effect compared to the estimation of the composite scores across all scenarios. Further research is needed to establish if this is an artefact of these parameters being estimated on different scales (i.e. the OKS ranges from 0 to 48, while the treatment effects observed in the trial were nonsignificant and close to zero), or whether this is a more generalisable finding.

This study only considers analysis scenarios with a single follow-up time point. This approach was chosen because the primary analyses of many trials focus on the primary endpoint at a specific follow-up time point, rather than analyses approaches that take into account the longitudinal data. Imputation of PROMs item level data at additional time points was ruled out as infeasible due to the low convergence rates already observed in the current scenarios. While including in the item level imputation model of PROMs follow-up data at intermediate time points may have improved imputations at the five-year follow-up, in practice this data is often less well collected at the outcome data at the primary follow-up time point. This leads to additional complexity of the imputation models, and was therefore not included in this study. Researchers should examine on a case-by-case basis if sufficient intermediate or later follow-up data is available to benefit the imputation of missing outcome data.

Missing not at random (MNAR) mechanism and misspecification of MI models were not considered in this paper, although Simons et al. [17] reported benefits of MI at the item level over MI at the score level for the latter scenario. However, it was felt that MI levels could be misspecified in a number of ways, and that the results from selected misspecifications may not be generalisable. This is because some variables are much more predictive of the missing data than others. The same applies to MNAR scenarios, which could be considered as misspecified MI models, as they are unable to account for important factors that are predictive of data being missing as well as the missing observations themselves. We recommend that MNAR analyses are best addressed as part of a sensitivity analysis [39–41].

Some of the non-convergence rates observed in the results are very high, and could have been improved by simplifying the MI models. However, MI models were constructed using the full base case datasets, and were then applied to all sample size scenarios to allow a direct comparison of performance between the different scenarios. In reality, MI models should be generated based on the dataset under consideration, and should adjust their complexity based on the type and quantity of data available, and ensure that relevant variables that are good predictors of data being missing, and/or the variables to be imputed, as well as that the functional form of the imputation model is appropriate for the data. Here, the correlations between outcomes and the covariates used in the imputation models were low to moderate. While this is representative of RCTs in general, the inclusion of more highly correlated variables will improve imputation results. Researchers should also run all required imputations within the same model. The approach chosen in this simulation study, whereby item level imputations were run one-by-one to exclude occasional instances of non-converges was chosen as a compromise to increase convergence rates within these simulations.

The high failure rates in some of the simulations may have resulted in a systematic selection bias being observed for the results of the relevant simulation scenarios, due to item MI being more likely to fail in datasets with certain characteristics. MI at the item level is considered less likely to be feasible in these scenarios, which were typically those with smaller sample sizes and higher missing data rates. More likely, however, is that for the smaller sample sizes, the ordinal logit models used in the item regression are of suboptimal fit to produce reliable prediction to inform the imputations. For this reason, imputation at the composite score or subscale level is recommended for these scenarios. Simulations with higher convergence rates are not thought to be affected.

Different numbers of imputations were used for the imputations at the item level, mainly for practical reasons including time taken to perform large numbers of the imputations at this level, and were therefore inconsistent across some of the scenarios. The number of imputations performed were still in line with available guidance, and are therefore expected to produce robust results. However, it may be possible that the differences in the number of imputations has added some variation to the study results.

Finally, simulations were restricted to 1000 iterations, again mainly for practical reasons including time taken to perform large numbers of the imputations at the item level. Additional simulations (up to 5000) were run for isolated scenarios, and results were consistent with those presented in this paper.

## Conclusions

We concluded that the differences between the imputation at the item/subscale level and the imputation at the composite score level are likely to be small across realistic settings in studies with incomplete patient-reported outcome measures.

In idealistic settings, the imputation at the item/subscale level may provide more precise estimates of treatment effect compared to the imputation at the composite score level or CCA, because it better captures the correlation amongst the different items.

However, both the case study and simulations suggested that the imputation at the item/subscale level is often infeasible and prone to convergence (perfect prediction) issues, and hence unlikely to be an appropriate method for imputing missing PROMs across more realistic circumstances.

Choosing an appropriate MI approach can help ensure the trial reports accurate estimates of treatment effects in the presence of missing data. However, better analytical approaches for handling missing data do not reduce the importance of taking active steps to minimising the occurrence of missing data at the trial’s design and follow-up stages. Appropriate sensitivity analysis to assess the impact of missing data on the trial results when changing the underlying assumptions about the missing data mechanism also remains imperative.

## Additional file

Additional file 1: Supplementary material for Multiple imputation for patient reported outcome measures in randomised controlled trials: advantages and disadvantages of imputing at the item, subscale or composite score level. **Table S1.** Overview of correlations between OKS outcome variables and covariates used in imputation models. **Table S2.** Overview of correlations between EQ-5D-3L outcome variables and covariates used in imputation models. **Table S3.** Overview of correlations between SF-12 outcome variables and covariates used in imputation models. **Figures S1-S9.** Supplementary graphs to Figs. 3, 4, 5, 6, 7, 8, 9, 10, 11 and 12 in the manuscript, showing mean absolute errors instead of root mean square errors. (DOCX 1613 kb)

## Abbreviations

CCA: Complete cases analysis
EQ-5D-3L: EuroQol 5 dimension 3-level questionnaire
IPW: Inverse probability weighting
KAT: Knee Arthroplasty Trial
MAE: Mean absolute error
MAR: Missing at random
MCS: Mental health component summary score
MI: Multiple imputation
MICE: Multiple imputation by chained equations
ML: Maximum Likelihood
MNAR: Missing not at random
OKS: Oxford knee score
PCI: Pain coping inventory
PCS: Physical health component summary score
PMM: Predicted mean matching
PROMs: Patient reported outcome measures
RCT: Randomised controlled trial
RMSE: Root mean square error
SE: Standard error
SF-12: 12-item short form survey
