# Correction to: multiple imputation for patient reported outcome measures in randomised controlled trials: advantages and disadvantages of imputing at the item, subscale or composite score level

**DOI:** 10.1186/s12874-018-0563-1

**Published:** 2018-10-16

**Authors:** Ines Rombach, Alastair M Gray, Crispin Jenkinson, David W Murray, Oliver Rivero-Arias

**Affiliations:** 1 Health Economics Research Centre, Nuffield Department of Population Health, University of Oxford, Oxford, UK; 2 Nuffield Department of Orthopaedics, Rheumatology and Musculoskeletal Sciences, University of Oxford, Oxford, UK; 3 Health Services Research Unit, Nuffield Department of Population Health, University of Oxford, Oxford, UK; 4 National Perinatal Epidemiology Unit, Nuffield Department of Population Health, University of Oxford, Oxford, UK

## Correction

Following publication of the original article [1], the authors reported that the following notation wasn’t used consistently. The original article has been corrected.

The correct notation is as follows:

- Unit-nonresponse
- Item-nonresponse
