# Supplementary material for: Multiple imputation for patient reported outcome measures in randomised controlled trials: advantages and disadvantages of imputing at the item, subscale or composite score level
Source: BMC Med Res Methodol. 2018 Aug 28;18:87. doi: 10.1186/s12874-018-0542-6 (PMC6114240; doi:10.1186/s12874-018-0542-6)
Supplement: Supplementary file 1 — Supplementary material for Multiple imputation for patient reported outcome measures in randomised controlled trials: advantages and disadvantages of imputing at the item, subscale or composite score level. Table S1. Overview of correlations between OKS outcome variables and covariates used in imputation models. Table S2. Overview of correlations between EQ-5D-3L outcome variables and covariates used in imputation models. Table S3. Overview of correlations between SF-12 outcome variables and covariates used in imputation models. Figures S1-S9. Supplementary graphs to Figs. 3, 4, 5, 6, 7, 8, 9, 10, 11 and 12 in the manuscript, showing mean absolute errors instead of root mean square errors. (DOCX 1613 kb) [file 12874_2018_542_MOESM1_ESM.docx]

**Table S1: Overview of correlations between OKS outcome variables and covariates used in imputation models**

|  | Follow-up OKS composite score | Follow-up OKS pain subscale | Follow-up OKS function subscale | Follow-up OKS item 1 | Follow-up OKS item 2 | Follow-up OKS item 3 | Follow-up OKS item 4 | Follow-up OKS item 5 | Follow-up OKS item 6 | Follow-up OKS item 7 | Follow-up OKS item 8 | Follow-up OKS item 9 | Follow-up OKS item 10 | Follow-up OKS item 11 | Follow-up OKS item 12 |
| --- | --- | --- | --- | --- | --- | --- | --- | --- | --- | --- | --- | --- | --- | --- | --- |
| Baseline OKS | 0.313 | 0.258 | 0.354 | 0.202 | 0.219 | 0.276 | 0.220 | 0.229 | 0.178 | 0.257 | 0.245 | 0.254 | 0.183 | 0.336 | 0.317 |
| Baseline OKS pain subscale | 0.253 | 0.227 | 0.261 | 0.176 | 0.148 | 0.214 | 0.187 | 0.199 | 0.161 | 0.197 | 0.236 | 0.205 | 0.162 | 0.247 | 0.227 |
| Baseline OKS function subscale | 0.337 | 0.254 | 0.414 | 0.202 | 0.274 | 0.310 | 0.225 | 0.229 | 0.169 | 0.291 | 0.214 | 0.275 | 0.179 | 0.394 | 0.380 |
| Baseline OKS item 1 | n/a | n/a | n/a | 0.130 | 0.097 | 0.146 | 0.128 | 0.097 | 0.068 | 0.157 | 0.141 | 0.132 | 0.084 | 0.168 | 0.134 |
| Baseline OKS item 2 | n/a | n/a | n/a | 0.182 | 0.318 | 0.275 | 0.191 | 0.208 | 0.154 | 0.178 | 0.189 | 0.240 | 0.156 | 0.330 | 0.296 |
| Baseline OKS item 3 | n/a | n/a | n/a | 0.175 | 0.205 | 0.292 | 0.165 | 0.212 | 0.157 | 0.214 | 0.200 | 0.221 | 0.142 | 0.256 | 0.266 |
| Baseline OKS item 4 | n/a | n/a | n/a | 0.078 | 0.098 | 0.117 | 0.129 | 0.092 | 0.056 | 0.106 | 0.115 | 0.117 | 0.073 | 0.156 | 0.144 |
| Baseline OKS item 5 | n/a | n/a | n/a | 0.115 | 0.114 | 0.167 | 0.124 | 0.171 | 0.077 | 0.142 | 0.144 | 0.119 | 0.077 | 0.165 | 0.140 |
| Baseline OKS item 6 | n/a | n/a | n/a | 0.031 | 0.015 | 0.031 | 0.044 | 0.019 | 0.093 | 0.050 | 0.054 | 0.024 | 0.048 | 0.045 | 0.058 |
| Baseline OKS item 7 | n/a | n/a | n/a | 0.090 | 0.096 | 0.158 | 0.116 | 0.128 | 0.090 | 0.314 | 0.094 | 0.131 | 0.057 | 0.207 | 0.226 |
| Baseline OKS item 8 | n/a | n/a | n/a | 0.177 | 0.086 | 0.170 | 0.117 | 0.182 | 0.125 | 0.155 | 0.274 | 0.170 | 0.111 | 0.155 | 0.138 |
| Baseline OKS item 9 | n/a | n/a | n/a | 0.164 | 0.162 | 0.221 | 0.191 | 0.209 | 0.157 | 0.197 | 0.184 | 0.231 | 0.155 | 0.265 | 0.238 |
| Baseline OKS item 10 | n/a | n/a | n/a | 0.128 | 0.129 | 0.157 | 0.151 | 0.152 | 0.154 | 0.131 | 0.163 | 0.166 | 0.189 | 0.212 | 0.209 |
| Baseline OKS item 11 | n/a | n/a | n/a | 0.176 | 0.216 | 0.233 | 0.185 | 0.165 | 0.140 | 0.209 | 0.156 | 0.237 | 0.181 | 0.379 | 0.290 |
| Baseline OKS item 12 | n/a | n/a | n/a | 0.109 | 0.155 | 0.180 | 0.161 | 0.129 | 0.076 | 0.158 | 0.149 | 0.168 | 0.106 | 0.240 | 0.311 |
| Baseline age | 0.067 | 0.068 | -0.080 | 0.081 | -0.010 | -0.065 | -0.007 | 0.092 | 0.044 | -0.024 | 0.148 | 0.011 | 0.018 | -0.119 | -0.086 |
| Gender | 0.131 | 0.057 | 0.219 | 0.047 | 0.030 | 0.099 | 0.071 | 0.022 | -0.024 | 0.292 | 0.084 | 0.089 | 0.046 | 0.217 | 0.172 |
| Height | 0.092 | 0.043 | 0.150 | 0.032 | 0.023 | 0.042 | 0.084 | -0.011 | -0.024 | 0.192 | 0.048 | 0.078 | 0.046 | 0.170 | 0.122 |
| Baseline ASA Grade | -0.111 | -0.075 | -0.148 | -0.034 | -0.085 | -0.088 | -0.102 | -0.069 | -0.040 | -0.133 | -0.050 | -0.086 | -0.070 | -0.122 | -0.153 |
| Size of recruting centre | 0.045 | 0.035 | 0.054 | 0.035 | 0.031 | 0.040 | 0.016 | 0.031 | 0.040 | 0.048 | 0.025 | 0.036 | 0.021 | 0.033 | 0.061 |
| Treatment allocation | 0.022 | 0.017 | 0.026 | 0.025 | 0.003 | 0.013 | -0.002 | 0.027 | 0.003 | 0.037 | 0.035 | 0.021 | -0.010 | 0.016 | 0.030 |

**Table S2: Overview of correlations between EQ-5D-3L outcome variables and covariates used in imputation models**

|  | Follow-up EQ-5D-3L index | Follow-up EQ-5D-3L Mobility | Follow-up EQ-5D-3L Self-care | Follow-up EQ-5D-3L Usual activities | Follow-up EQ-5D-3L Pain | Follow-up EQ-5D-3L Anxiety/ depression |
| --- | --- | --- | --- | --- | --- | --- |
| Baseline EQ-5D-3L index | 0.246 | -0.157 | -0.190 | -0.181 | -0.186 | -0.191 |
| Baseline EQ-5D-3L Mobility | n/a | 0.065 | 0.030 | 0.041 | 0.030 | 0.008 |
| Baseline EQ-5D-3L Self-care | n/a | 0.181 | 0.356 | 0.216 | 0.137 | 0.155 |
| Baseline EQ-5D-3L Usual activities | n/a | 0.148 | 0.116 | 0.184 | 0.124 | 0.107 |
| Baseline EQ-5D-3L Pain | n/a | 0.111 | 0.126 | 0.124 | 0.167 | 0.123 |
| Baseline EQ-5D-3L Anxiety/ depression | n/a | 0.144 | 0.108 | 0.156 | 0.100 | 0.318 |
| Baseline age | 0.016 | 0.066 | 0.022 | 0.078 | -0.046 | -0.037 |
| Gender | 0.116 | -0.110 | -0.099 | -0.129 | -0.063 | -0.142 |
| Height | 0.076 | -0.072 | -0.071 | -0.090 | -0.049 | -0.096 |
| Baseline ASA Grade | 0.054 | -0.046 | -0.003 | -0.009 | -0.053 | -0.001 |
| Size of recruiting centre | 0.017 | -0.032 | -0.001 | -0.041 | -0.020 | -0.011 |
| Treatment allocation | 0.032 | -0.024 | -0.022 | -0.034 | -0.007 | -0.030 |

**Table S3: Overview of correlations between SF-12 outcome variables and covariates used in imputation models**

|  | Follow-up SF-12 PCS | Follow-up SF-12 MCS | Follow-up SF-12 Q1 | Follow-up SF-12 Q2a | Follow-up SF-12 Q2b | Follow-up SF-12 Q3a | Follow-up SF-12 Q3b | Follow-up SF-12 Q4a | Follow-up SF-12 Q4b | Follow-up SF-12 Q5 | Follow-up SF-12 Q6a | Follow-up SF-12 Q6b | Follow-up SF-12 Q6c | Follow-up SF-12 Q7 |
| --- | --- | --- | --- | --- | --- | --- | --- | --- | --- | --- | --- | --- | --- | --- |
| Baseline SF-12 PCS | 0.309 | 0.024 | 0.207 | 0.244 | 0.250 | 0.278 | 0.247 | 0.078 | 0.087 | 0.201 | 0.047 | 0.166 | 0.041 | 0.151 |
| Baseline SF-12 MCS | 0.196 | 0.422 | 0.294 | 0.193 | 0.216 | 0.249 | 0.240 | 0.363 | 0.324 | 0.237 | 0.328 | 0.348 | 0.327 | 0.323 |
| Baseline SF-12 Q1 | n/a | n/a | 0.476 | 0.249 | 0.235 | 0.299 | 0.280 | 0.245 | 0.250 | 0.261 | 0.260 | 0.341 | 0.214 | 0.285 |
| Baseline SF-12 Q2a | n/a | n/a | 0.148 | 0.217 | 0.195 | 0.215 | 0.186 | 0.136 | 0.138 | 0.149 | 0.094 | 0.145 | 0.102 | 0.162 |
| Baseline SF-12 Q2b | n/a | n/a | 0.161 | 0.196 | 0.254 | 0.198 | 0.174 | 0.130 | 0.125 | 0.159 | 0.088 | 0.164 | 0.074 | 0.149 |
| Baseline SF-12 Q3a | n/a | n/a | 0.198 | 0.230 | 0.255 | 0.281 | 0.265 | 0.190 | 0.177 | 0.230 | 0.114 | 0.200 | 0.144 | 0.192 |
| Baseline SF-12 Q3b | n/a | n/a | 0.173 | 0.212 | 0.215 | 0.262 | 0.265 | 0.150 | 0.143 | 0.188 | 0.142 | 0.191 | 0.130 | 0.177 |
| Baseline SF-12 Q4a | n/a | n/a | 0.213 | 0.143 | 0.144 | 0.177 | 0.182 | 0.284 | 0.243 | 0.168 | 0.205 | 0.215 | 0.229 | 0.242 |
| Baseline SF-12 Q4b | n/a | n/a | 0.240 | 0.153 | 0.157 | 0.202 | 0.198 | 0.314 | 0.288 | 0.196 | 0.197 | 0.255 | 0.261 | 0.260 |
| Baseline SF-12 Q5 | n/a | n/a | 0.149 | 0.193 | 0.214 | 0.257 | 0.216 | 0.122 | 0.101 | 0.218 | 0.081 | 0.151 | 0.106 | 0.173 |
| Baseline SF-12 Q6a | n/a | n/a | 0.245 | 0.160 | 0.176 | 0.194 | 0.144 | 0.267 | 0.241 | 0.205 | 0.332 | 0.269 | 0.269 | 0.226 |
| Baseline SF-12 Q6b | n/a | n/a | 0.336 | 0.265 | 0.301 | 0.327 | 0.278 | 0.244 | 0.224 | 0.252 | 0.222 | 0.414 | 0.187 | 0.254 |
| Baseline SF-12 Q6c | n/a | n/a | 0.222 | 0.146 | 0.203 | 0.187 | 0.196 | 0.279 | 0.241 | 0.191 | 0.293 | 0.242 | 0.288 | 0.237 |
| Baseline SF-12 Q7 | n/a | n/a | 0.184 | 0.224 | 0.226 | 0.269 | 0.272 | 0.253 | 0.238 | 0.209 | 0.193 | 0.254 | 0.207 | 0.301 |
| Baseline age | -0.118 | -0.024 | -0.035 | -0.166 | -0.123 | -0.117 | -0.124 | -0.072 | -0.090 | -0.014 | 0.056 | -0.110 | 0.018 | -0.108 |
| Gender | 0.098 | 0.102 | 0.102 | 0.116 | 0.159 | 0.089 | 0.075 | 0.064 | 0.071 | 0.042 | 0.136 | 0.143 | 0.088 | 0.085 |
| Height | 0.065 | 0.098 | 0.085 | 0.073 | 0.126 | 0.063 | 0.043 | 0.066 | 0.080 | 0.041 | 0.119 | 0.113 | 0.086 | 0.056 |
| Baseline ASA Grade | 0.043 | -0.013 | 0.023 | 0.001 | 0.011 | 0.032 | 0.063 | 0.017 | 0.010 | 0.041 | 0.007 | 0.021 | -0.053 | 0.002 |
| Size of recruiting centre | 0.013 | 0.066 | 0.005 | 0.000 | 0.020 | 0.036 | 0.035 | 0.049 | 0.055 | 0.049 | 0.058 | 0.068 | 0.051 | 0.018 |
| Treatment allocation | 0.015 | 0.035 | -0.005 | 0.009 | 0.032 | 0.029 | 0.008 | 0.009 | 0.012 | 0.029 | 0.040 | 0.059 | 0.039 | 0.017 |


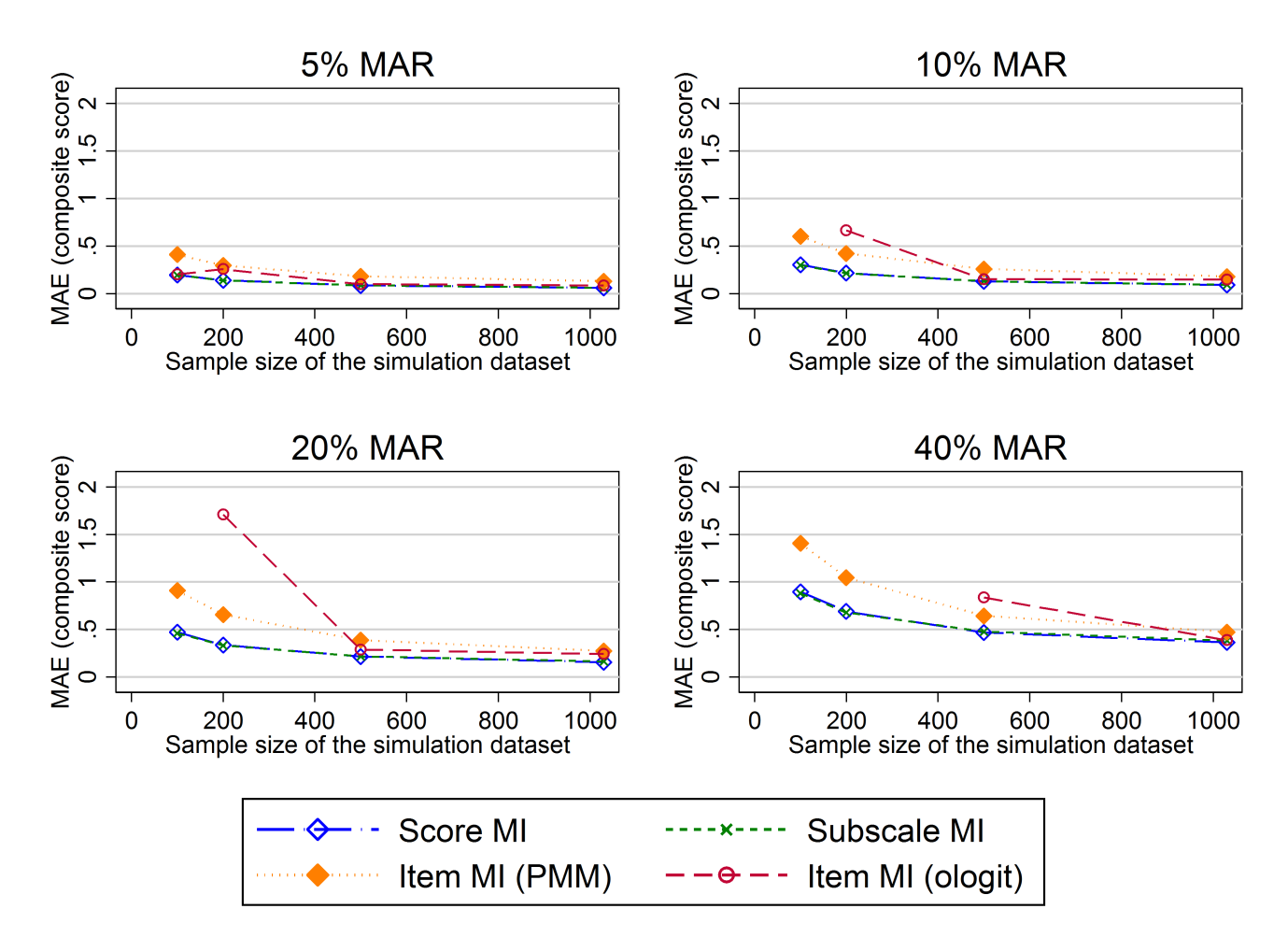


Figure S1: MAE in the OKS composite score estimates (observed missing data patterns)

Abbreviations: MAE – Mean absolute error; MAR – Missing at random; MI – Multiple imputation; OKS –Oxford knee score; PMM – Predicted mean matching.


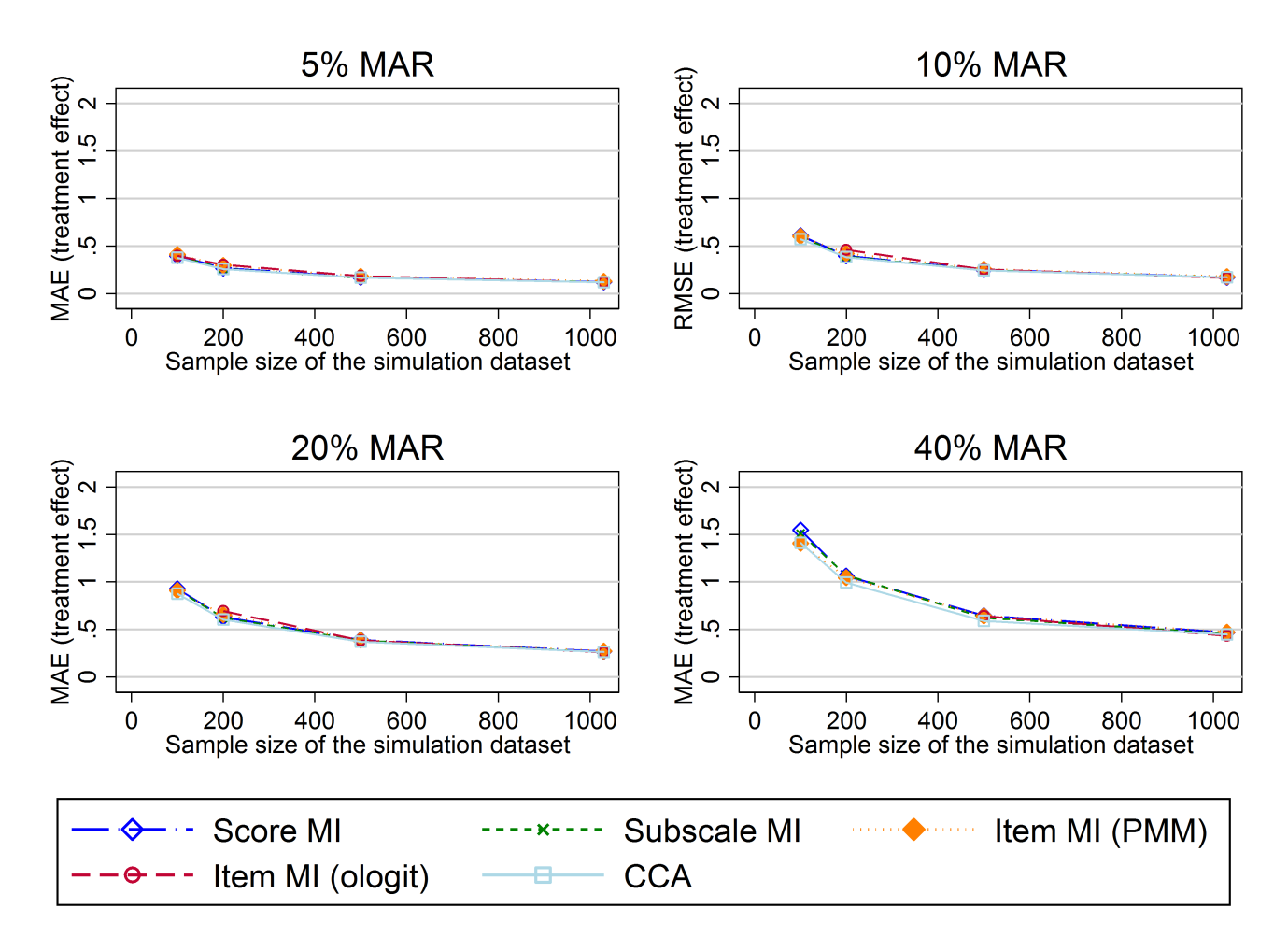


Figure S2: MAE in the treatment effect estimates using the imputed OKS composite scores as the outcome variable in the regression model (observed missing data pattern)

Abbreviations: CCA – Complete cases analysis; MAE – Mean absolute error; MAR – Missing at random; MI – Multiple imputation; OKS –Oxford knee score; PMM – Predicted mean matching.


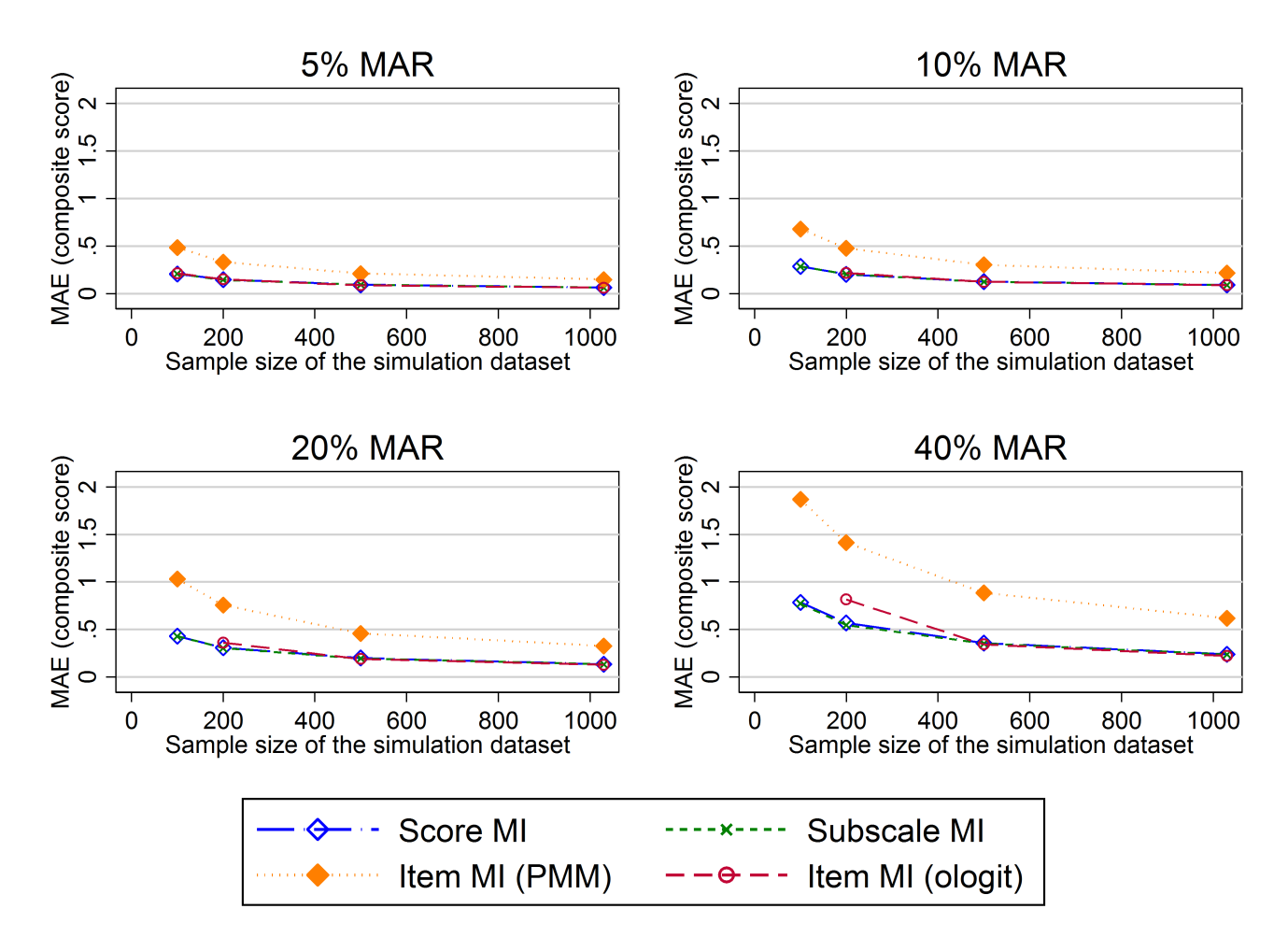


Figure S3: MAE in the OKS composite score estimates (unit-nonresponse)

Abbreviations: MAE – Mean absolute error; MAR – Missing at random; MI – Multiple imputation; OKS –Oxford knee score; PMM – Predicted mean matching.


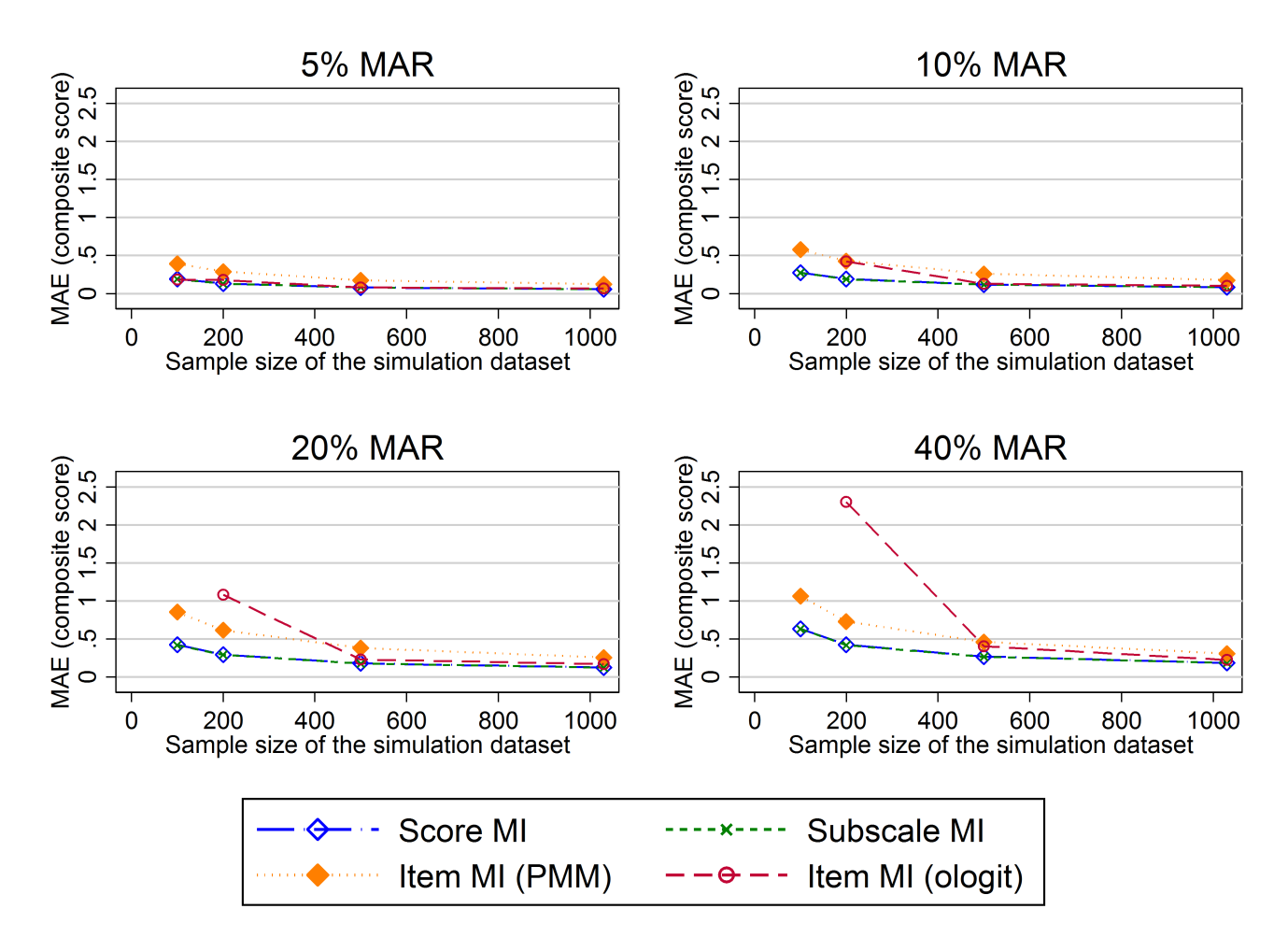


Figure S4: MAE in the OKS composite score estimates (70% item non-response simulations)

Abbreviations: MAE – Mean absolute error; MAR – Missing at random; MI – Multiple imputation; OKS –Oxford knee score; PMM – Predicted mean matching.


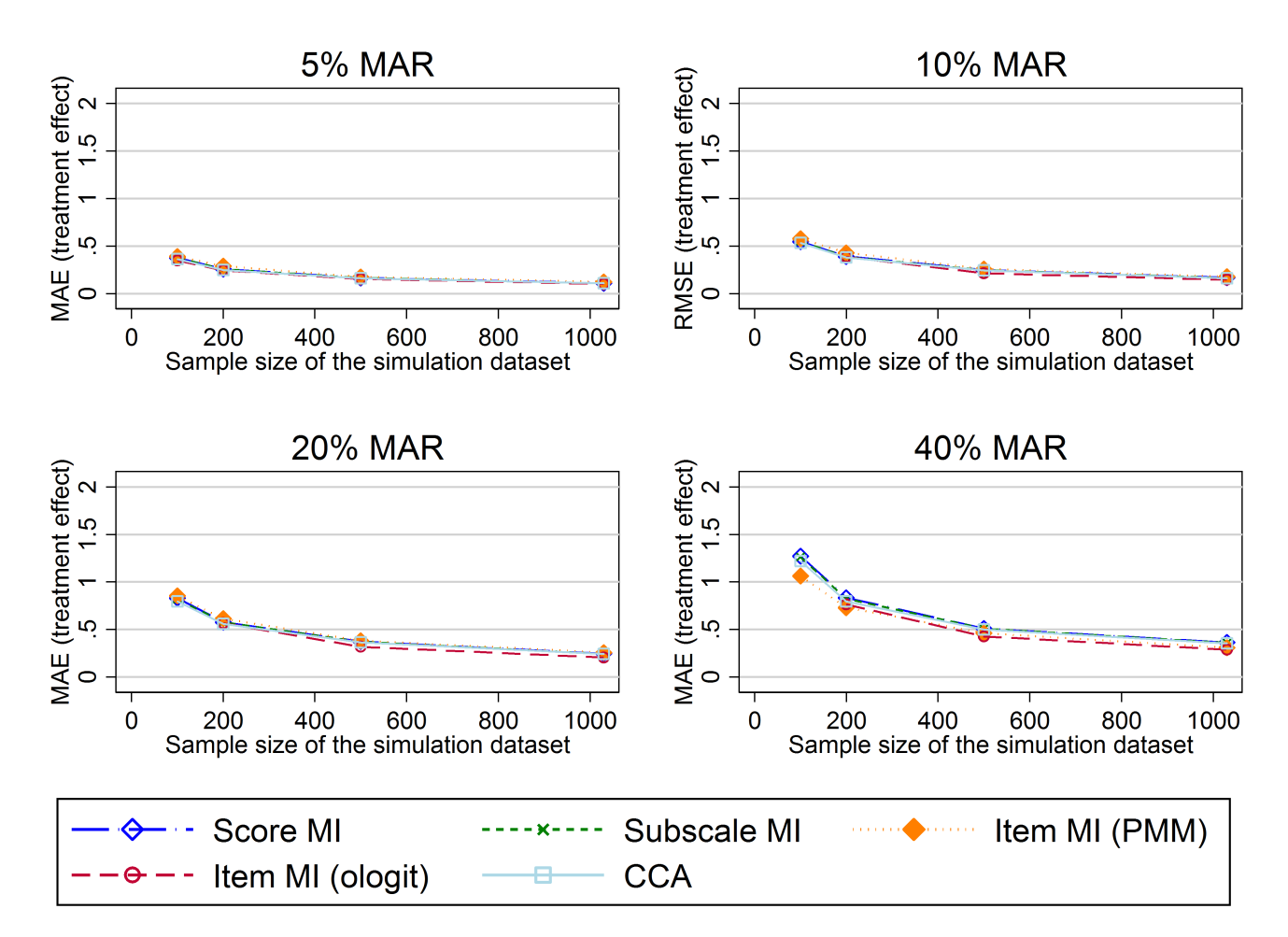


Figure S5: MAE in the treatment coefficient estimates using the imputed OKS composite scores as the outcome variable in the regression model (70% item-nonresponse)

Abbreviations: CCA – Complete cases analysis; MAE – Mean absolute error; MAR – Missing at random; MI – Multiple imputation; OKS –Oxford knee score; PMM – Predicted mean matching.


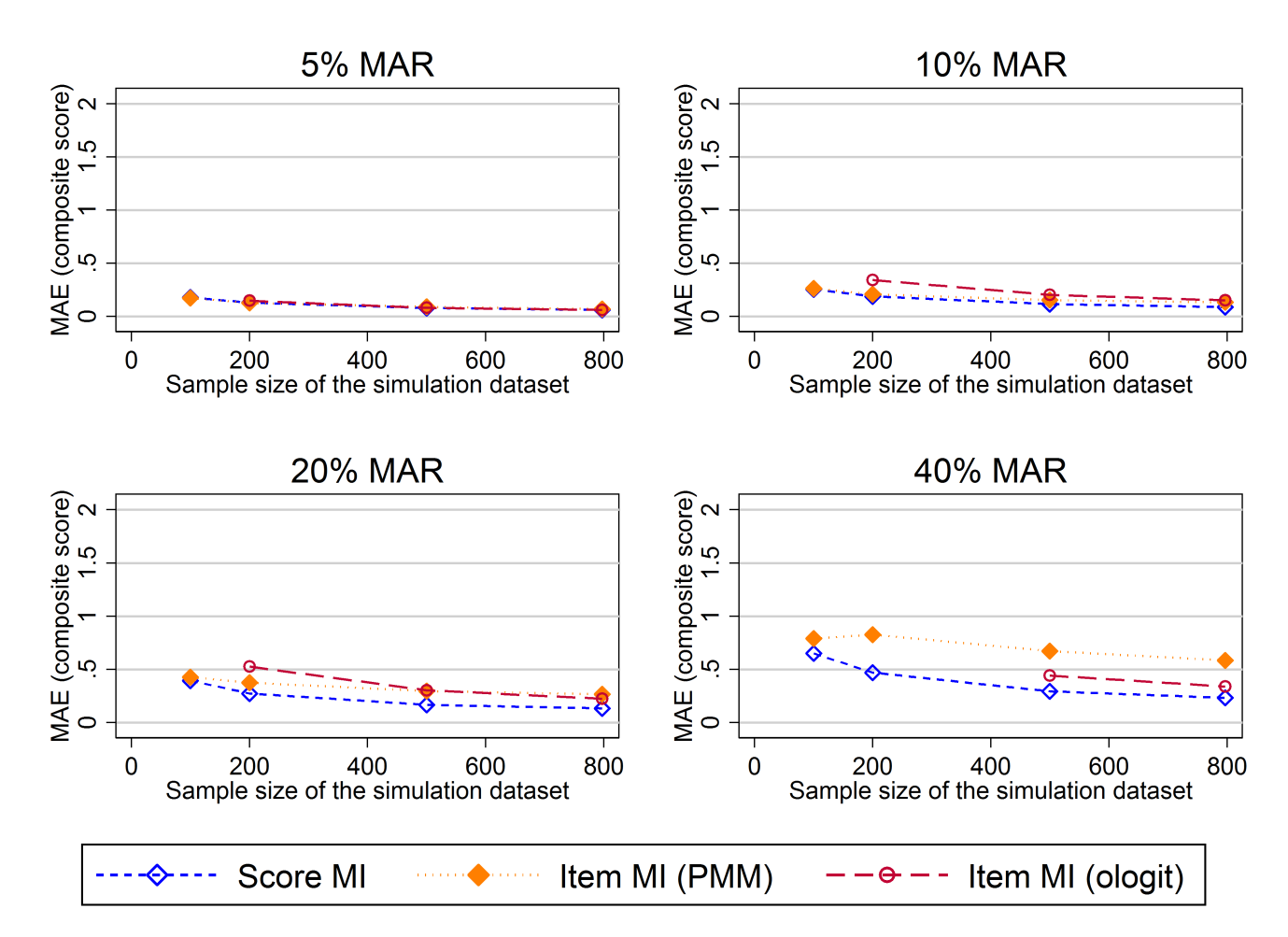


Figure S6: MAE in the MCS composite score estimates

Abbreviations: MAE – Mean absolute error; MAR – Missing at random; MI – Multiple imputation; MCS –SF-12 Mental component summary score; PMM – Predicted mean matching.


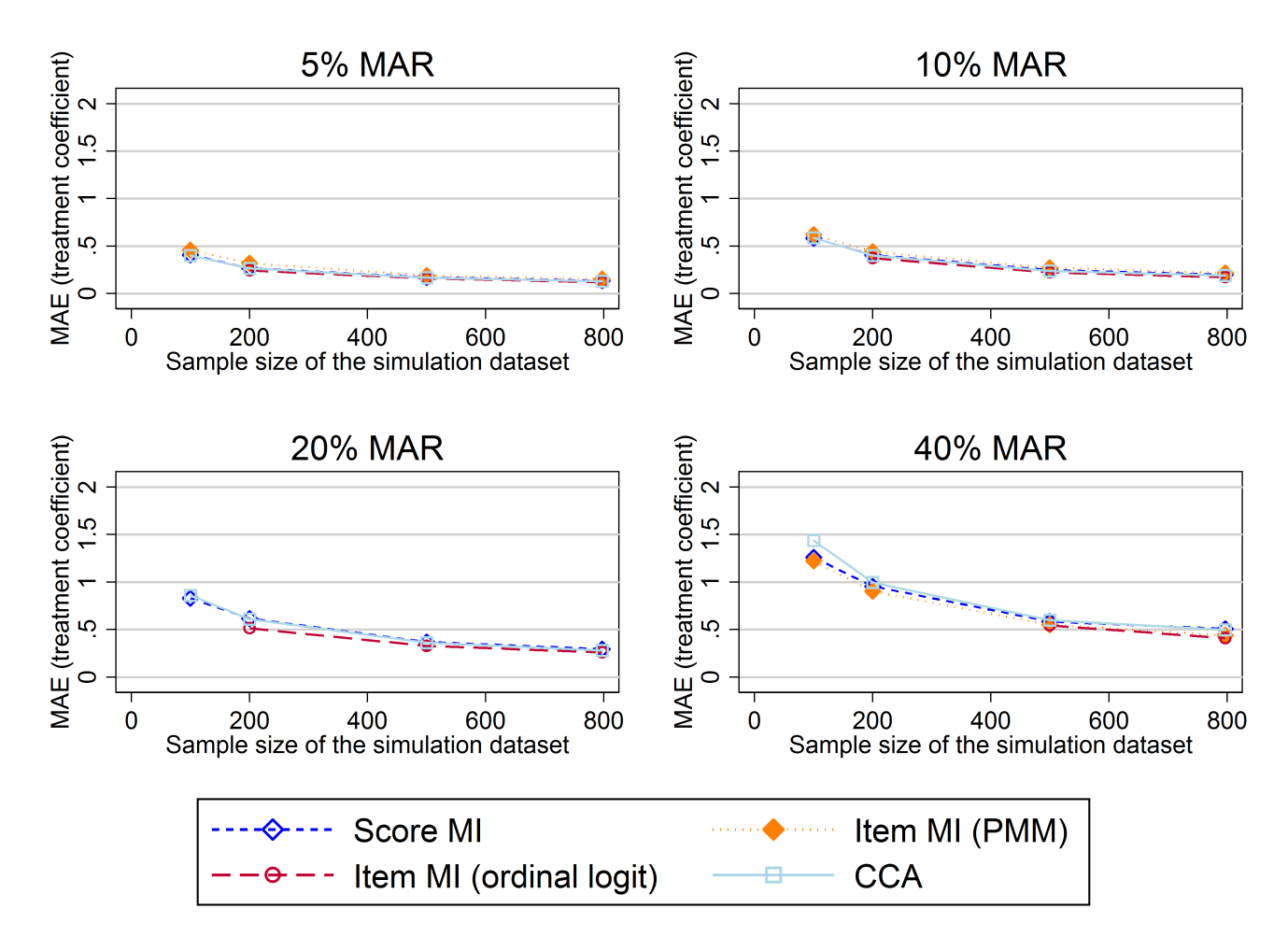


Figure S7: MAE in the treatment coefficient estimates using the imputed PCS composite scores as the outcome variable in the regression model

Abbreviations: CCA – Complete cases analysis; MAE – Mean absolute error; MAR – Missing at random; MI – Multiple imputation; PCS – Physical component summary score; PMM – Predicted mean matching.


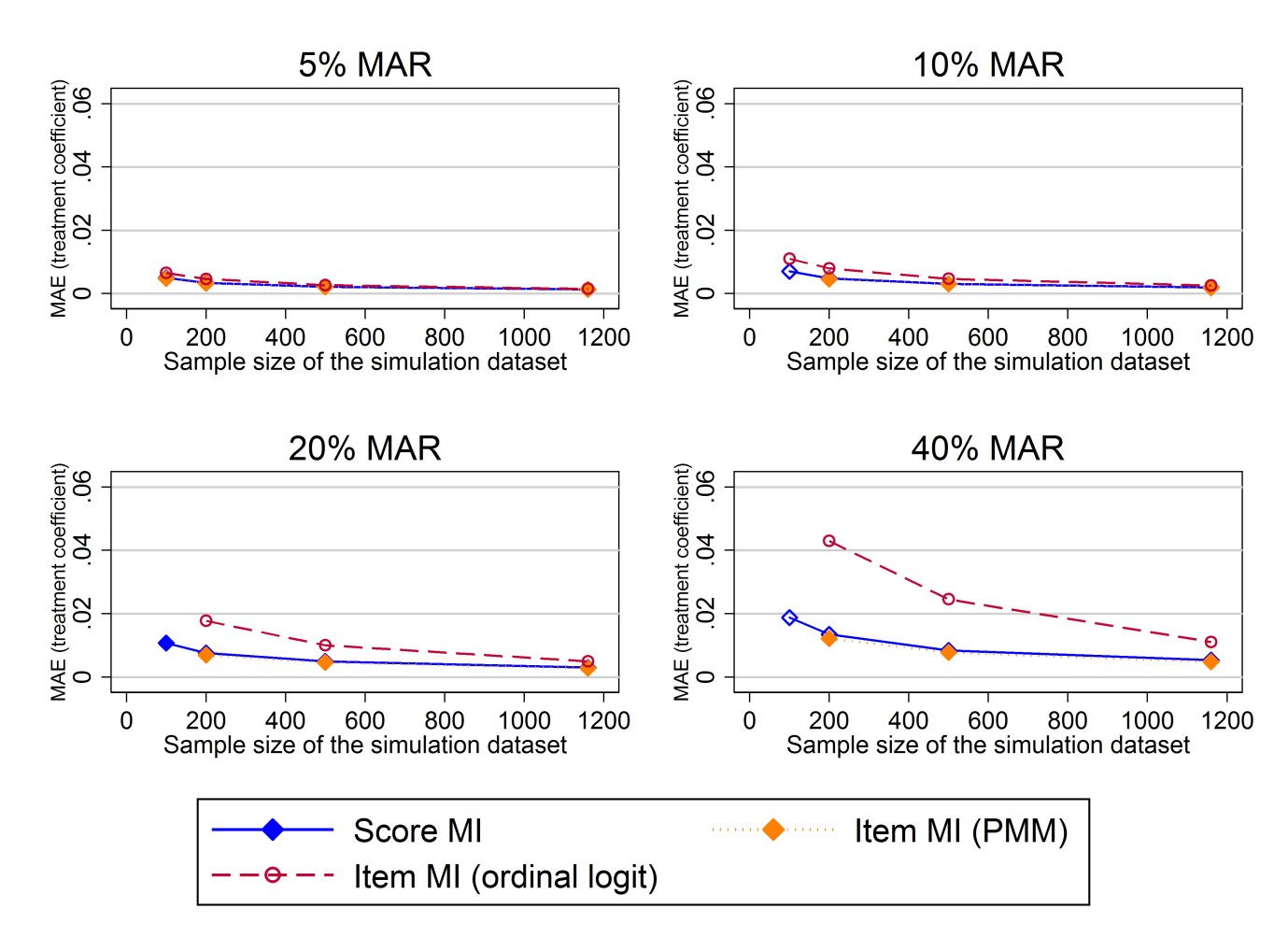


Figure S8: MAE in the EQ-5D-3L composite score estimates

Abbreviations: EQ-5D-3L - EuroQol 5 dimension 3-level questionnaire; MAE – Mean absolute error; MAR – Missing at random; MI – Multiple imputation; MCS –SF-12 Mental component summary score; PMM – Predicted mean matching.


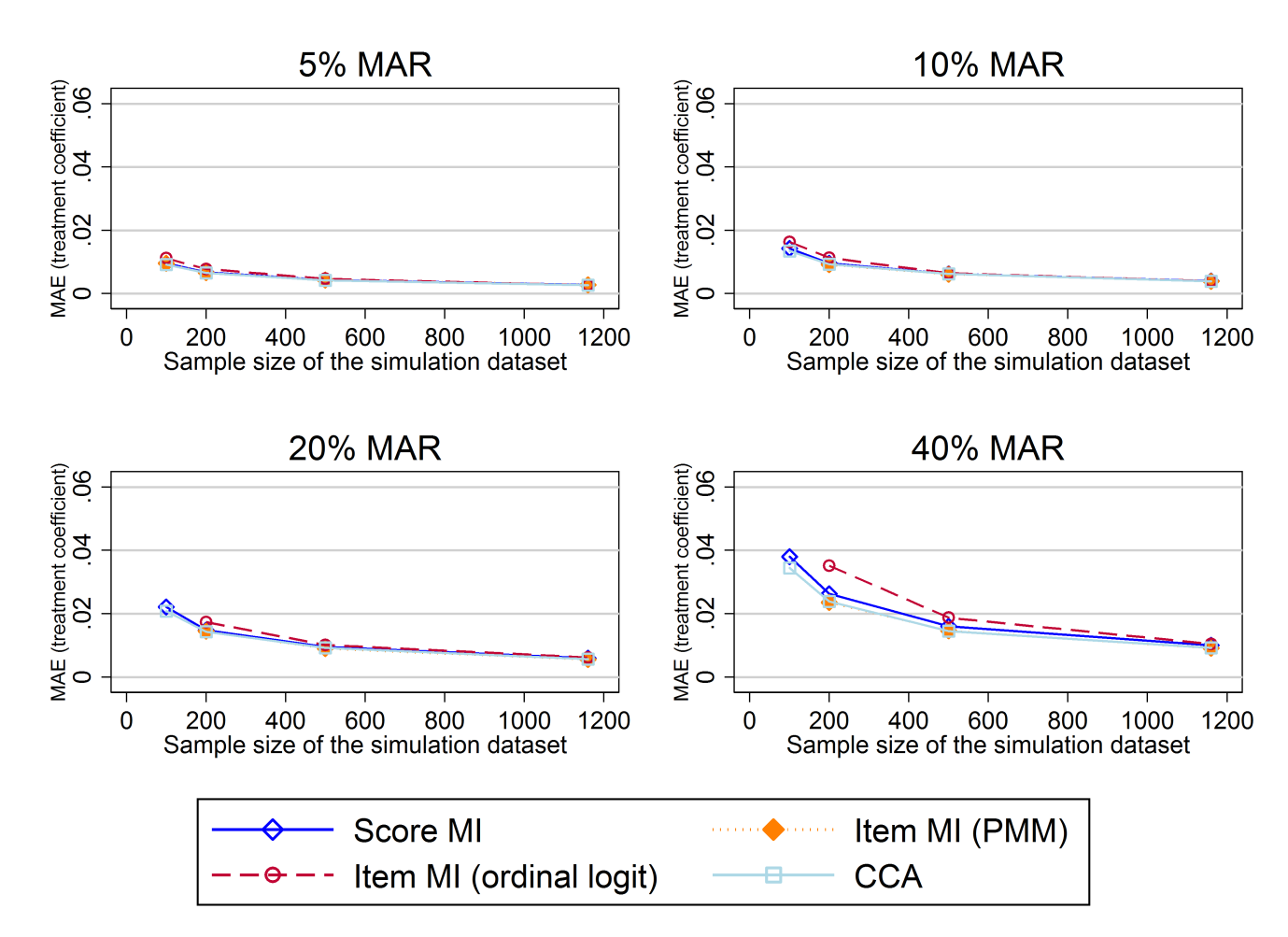


Figure S9: MAE in the treatment coefficient estimates using the imputed EQ-5D-3L composite scores as the outcome variable in the regression model

Abbreviations: CCA – Complete cases analysis; EQ-5D-3L - EuroQol 5 dimension 3-level questionnaire; MAE – Mean absolute error; MAR – Missing at random; MI – Multiple imputation; PMM – Predicted mean matching.
